# Epidemiological Trends and Treatment Outcomes: Findings of a TB Survey From Selected Districts of Madhya Pradesh, India

**DOI:** 10.7759/cureus.84663

**Published:** 2025-05-23

**Authors:** Manohar Bhatia, Yogesh Sharma, Vikas Dwivedi, Pradeep Sukla, Bikramjeet Mitra, Vikas Pandey, Varsha Rai

**Affiliations:** 1 Community Medicine, Government Medical College, Datia, Datia, IND; 2 Community Medicine, World Health Organization Regional Office, Bhopal, IND; 3 Community Medicine, Chhindwara Institute of Medical Sciences, Chhindwara, IND; 4 Biostatistics, Government Medical College, Datia, Datia, IND; 5 Tuberculosis and Infectious Diseases, State TB Task Force, Bhopal, IND

**Keywords:** epidemiological trends, private sector notification, tb mortality, treatment outcomes, tuberculosis

## Abstract

Background and objective: Tuberculosis (TB) remains a significant public health challenge in India, particularly in Madhya Pradesh. In this study, we aimed to examine epidemiological trends and treatment outcomes in TB patients in the Datia and Tikamgarh districts of Madhya Pradesh, from 2018 to 2022, to inform targeted TB control strategies.

Methods: We conducted a retrospective observational study using data from the National TB Elimination Program (NTEP), accessed through the Nikshay portal (a Government of India initiative). We analyzed trends in TB notifications, rates of microbiological confirmation, treatment outcomes, and co-infections. Statistical tests, including the Mann-Whitney U test, Kruskal-Wallis test, and chi-square test, were employed, with a p-value of less than 0.05 indicating statistical significance.

Results: In Datia, the proportion of pediatric TB cases decreased from 6% to 3% (p = 0.04), while extrapulmonary TB (EPTB) cases rose from 11.3% to 13.8% (p = 0.02). Notifications from the private sector significantly increased from 4% to 28% (p = 0.03), whereas drug-resistant TB (DR-TB) cases fell from 2% to 1% (p = 0.02). TB-related mortality rose from 3.28% to 3.93% (p = 0.008), with the proportion of patients lost to follow-up remaining stable at 9%-10% (p = 0.02).

In Tikamgarh, pediatric TB rates declined from 7.7% to 6.3% (p = 0.04), and EPTB cases increased from 4.77% to 9.37% (p = 0.02). Notifications from the private sector surged from 1.13% to 20.75% (p = 0.03). DR-TB rates decreased from 4.33% to 1% (p = 0.02), but TB-related mortality increased from 1.87% to 5.46% (p = 0.008). The rate of patients lost to follow-up improved slightly, decreasing from 12.71% to 10.09% (p = 0.02).

Conclusion: The reduction in pediatric TB and DR-TB indicates progress in diagnosis and treatment adherence. However, the rising incidence of EPTB and increasing mortality rates highlight ongoing challenges. Enhancing private sector involvement, improving patient adherence, and integrating HIV-TB care are crucial for achieving India’s TB elimination objectives.

## Introduction

Tuberculosis (TB) remains a significant public health concern in India, contributing substantially to the global TB burden. Despite efforts to control its spread, India continues to report a high incidence of TB, with regional variations influenced by socioeconomic and environmental factors. Madhya Pradesh, a state of central India, has consistently recorded high TB prevalence rates, necessitating an assessment of epidemiological trends and treatment outcomes in selected districts to inform targeted intervention strategies [1].

Epidemiological surveillance is crucial for understanding TB transmission dynamics, risk factors, and treatment adherence. Studies have shown a higher TB prevalence among marginalized communities, individuals with limited healthcare access, and those with occupational exposure to bioaerosols and pollutants [2,3]. The co-existence of non-communicable diseases (NCDs) further exacerbates TB outcomes, increasing the risk of complications and mortality [4]. Early detection through active case finding and household contact screening has proven effective in identifying undiagnosed TB cases in high-burden areas [5,6]. However, stigma, lack of awareness, and reluctance to disclose disease status continue to impede timely diagnosis and treatment adherence [7].

Treatment adherence is critical for TB control success. The Directly Observed Treatment, Short-Course (DOTS) strategy has been a cornerstone of India's TB control program, yet dropout rates and incomplete adherence remain significant challenges [8]. The implementation of tuberculosis preventive therapy (TPT) for latent TB infection (LTBI) has shown promising results in reducing disease progression among household contacts, underscoring the need for programmatic expansion [9].

To address disparities, India launched the Sub-National Certification (SNC) initiative in 2021 to incentivize TB control efforts. SNC involves independent verification by agencies such as Indian Association of Preventive & Social Medicine (IAPSM), Indian Council of Medical Research - National Institute of Epidemiology (ICMR-NIE), and WHO, with assessment criteria including a ≥20% decline in TB drug consumption, a ≥20% increase in the number needed to test (NNT) since 2015, and an 80% TB score achievement. High-performing districts receive recognition and financial incentives [10].

This study aimed to evaluate the epidemiological patterns and treatment outcomes of TB patients in selected districts of Madhya Pradesh. By analyzing TB incidence, treatment completion rates, and associated risk factors, the findings seek to contribute to India’s National Strategic Plan for Tuberculosis Elimination by 2025 by identifying regional gaps and informing targeted interventions to strengthen TB control efforts at the sub-national level.

## Materials and methods

Study design

This was a retrospective observational study conducted to assess epidemiological trends and treatment outcomes of TB in the Datia and Tikamgarh districts of Madhya Pradesh, India. These districts were chosen to capture contrasting TB burden and healthcare infrastructure. We included all TB cases reported between January 2018 and December 2022 from the Nikshay portal [11] in compliance with National TB Elimination Program (NTEP) guidelines.

Study population and data collection

The study population included every individual registered with Nikshay and under treatment in Datia and Tikamgarh during the study period. After securing approval from the District TB Offices of Tikamgarh and Niwari, we extracted data from the Nikshay portal, in accordance with NTEP confidentiality guidelines. Collected variables included demographic profile, TB classification (pulmonary vs. extrapulmonary); diagnostic modality, including smear microscopy, Xpert MTB/RIF (Cepheid, Sunnyvale, USA), and culture; drug resistance profile; treatment outcome; notifications from the private sector; HIV co-infection status and pediatric TB incidence. Incomplete or missing records were excluded from the data.

Variables and definitions

Notification trends included total number of cases, proportions of pediatric (<15 years) and extrapulmonary TB (EPTB) cases, and private sector notifications. Microbiological confirmation included percentage of diagnoses established by smear microscopy, Xpert MTB/RIF, or culture. Drug resistance meant prevalence of multidrug-resistant TB (MDR-TB) and rifampicin-resistant TB. Treatment outcomes included rates of treatment success, mortality, loss to follow-up, and regimen modifications. HIV-TB co-infection included proportion of TB patients concurrently diagnosed with HIV.

Statistical analysis

Data were analyzed using jamovi v2.3.28. Categorical variables were compared using the chi-square test, with statistical significance defined as p < 0.05.

## Results

Trends in TB case notifications in Datia (2018-2022)

**Figure 1:**
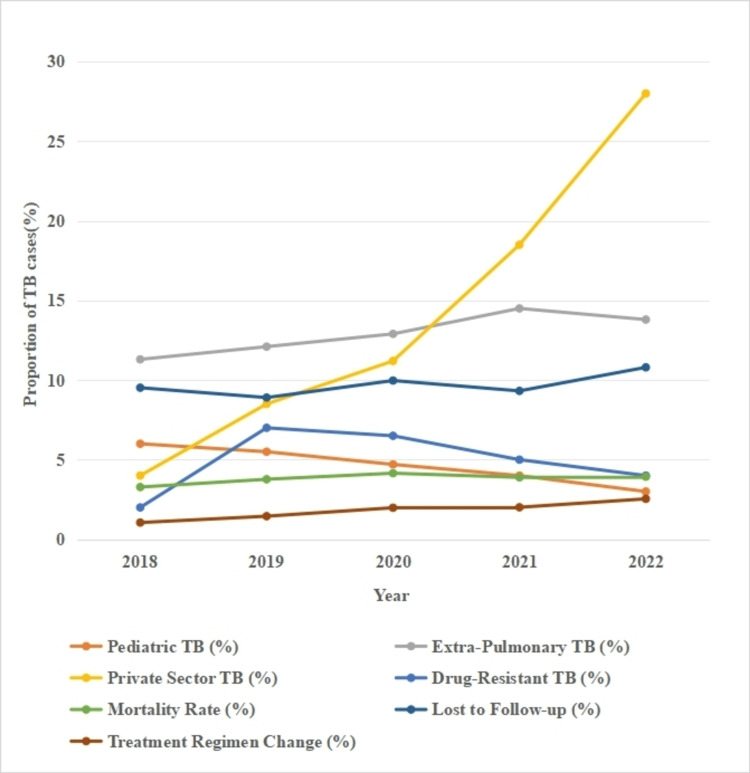
Trends in TB case notifications in Datia

Between 2018 and 2022, Datia district witnessed notable shifts in several TB indicators. Pediatric TB notifications declined by half from 6% to 3% (Figure 1). In contrast, the proportion of extrapulmonary TB cases increased from 11.3% to 13.8% over the same period. Private sector case notifications surged markedly, from 4% in 2018 to 28% in 2022, showing the improved engagement of private providers. The proportion of drug-resistant TB (DR-TB) cases decreased from 2% to 1%, whereas TB mortality increased from 3.28% to 3.93%. The proportion of patients lost to follow-up remained at around 9%-10%, and changes in the treatment regimens of TB patients increased more than double from 1.05% to 2.54% (Figure 1).

Trends in TB case notifications in Tikamgarh (2018-2022)

**Figure 2:**
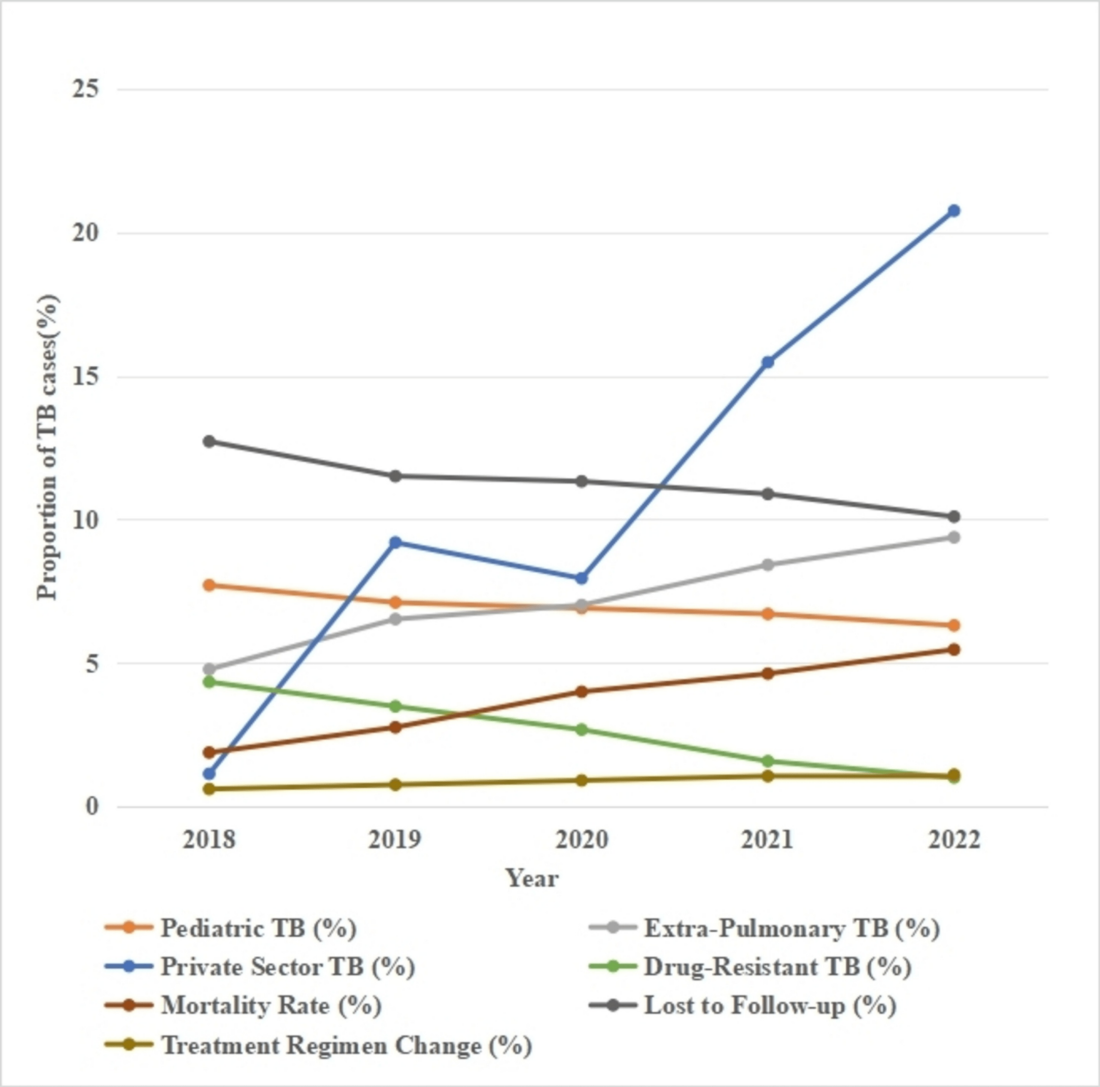
Trends in TB case notifications in Tikamgarh (2018-2022)

In the Tikamgarh district, pediatric TB rates decreased from 7.7% in 2018 to 6.3% in 2022, mirroring the downward trend seen in Datia (Figure 2). Extrapulmonary TB increased substantially from 4.77% to 9.37%, and private sector notifications increased from 1.13% to 20.75%. The proportion of DR-TB cases decreased from 4.33% to 1, while TB-related mortality increased from 1.87% to 5.46%. The proportion of TB cases lost to follow-up declined from 12.71% to 10.09%, and treatment regimen changes increased from 0.6% to 1.1%, indicating better clinical management and the addition of newer regimens for treatment (Figure 2).

Notification and diagnostic patterns

The proportion of pediatric TB notifications remained consistently higher in Datia compared to Tikamgarh (p = 0.04) (Table 1). Both districts experienced significant increases in extrapulmonary TB over the study period; however, the rise was more pronounced in Tikamgarh (p = 0.02). Microbiological confirmation rates exhibited notable fluctuations: Datia improved to 60.73% by 2022, whereas Tikamgarh’s rate declined to 24.4% in 2021 before rebounding (p = 0.01). Likewise, private sector notifications rose steadily in both settings, with Tikamgarh demonstrating a more rapid escalation (p = 0.03).

**Table 1:** Trends in TB notifications and distribution of cases in Datia and Tikamgarh from 2018 to 2022 *p<0.05, using chi-square test

Variables	Datia					Tikamgarh					p-value
	2018	2019	2020	2021	2022	2018	2019	2020	2021	2022	
Pediatric TB (%)	7.7	7.1	6.9	6.7	6.3	6	5.5	4.7	4	3	0.04*
Extra-pulmonary TB (%)	4.77	6.52	7.01	8.41	9.37	11.3	12.1	12.9	14.5	13.8	0.02*
Microbiologically confirmed TB (%)	56.59	52.19	55.9	53.15	60.73	46.46	41.25	38.6	24.4	46.5	0.01*
Private sector TB (%)	1.13	9.19	7.94	15.47	20.75	4	8.5	11.2	18.5	28	0.03*
Drug-resistant TB (%)	4.33	3.48	2.67	1.57	1	2	7	6.5	5	4	0.02*

Treatment outcomes, mortality, and follow-up

Drug-resistant TB prevalence remained higher in Tikamgarh than in Datia throughout the study, although both districts saw significant declines (p = 0.02) (Table 2). Treatment success rates decreased in both areas, reaching a low of 75.65% in Datia in 2020, with a partial rebound by 2022 (p = 0.005). Mortality associated with TB in Datia doubled over the five-year period to 5.46% in 2022, while Tikamgarh’s death rate remained relatively stable (p = 0.008). Rates of loss to follow-up improved slightly in Datia but continued to exceed those in Tikamgarh (p = 0.02). Finally, modifications to treatment regimens and patient transfer-out events occurred significantly more often in Tikamgarh than in Datia (p = 0.04, for both indicators) (Table 2).

**Table 2:** Distribution of cases based on treatment outcomes and mortality rate in Datia and Tikamgarh from 2018 to 2022 *p<0.05, **p<0.01, using chi-square test

Variables	Datia					Tikamgarh					p-value
	2018	2019	2020	2021	2022	2018	2019	2020	2021	2022	
Treatment success rate (%)	83.07	81.34	75.65	77.88	79.44	84.34	81.89	70.21	74.55	83.01	0.005**
Mortality rate (%)	1.87	2.75	3.99	4.62	5.46	3.28	3.77	4.15	3.89	3.93	0.008**
Lost to follow-up (%)	12.71	11.5	11.32	10.88	10.09	9.52	8.9	9.97	9.32	10.8	0.02*
Treatment regimen change (%)	0.6	0.75	0.9	1.05	1.1	1.05	1.45	1.98	2.01	2.54	0.04*

HIV-TB co-infection trends

Although overall HIV-TB co-infection rates were low, Datia exhibited a gradual upward trend that approached statistical significance by 2022 (p = 0.07).

## Discussion

We found that TB trends in Datia and Tikamgarh (2018-2022) were consistent with national studies, particularly regarding pediatric TB, EPTB, private sector notifications, DR-TB, mortality rates, treatment success, and HIV-TB co-infection.

Pediatric TB cases have decreased in Datia (from 6% to 3%) and Tikamgarh (from 7.7% to 6.3%), reflecting improved case detection. Similarly, Jogdand et al., in 2024, reported an increase in TB notifications in Solapur, from 58.15 per 100,000 in 2015 to 90.69 in 2022, following the mandatory notification policy [12]. The rise in private sector notifications in Datia (from 4% to 28%) and Tikamgarh (from 1.13% to 20.75%) aligns with findings of Siddaiah et al. (2019), who discovered that only 23.2% of TB cases in a Bengaluru hospital were notified, citing barriers such as misconceptions and lack of awareness [13].

Conversely, EPTB cases increased in Datia (from 11.3% to 13.8%) and Tikamgarh (from 4.77% to 9.37%). A study reported that 18% of 3,972 EPTB cases tested positive, with pleural TB (31%) being the most common [14]. However, this study noted a declining EPTB trend post-2018, with a slight rise in 2022, which may be attributed to regional variations in case detection and access to diagnostics.

DR-TB cases declined in Datia (from 2% to 1%) and Tikamgarh (from 4.33% to 1%). The effectiveness of bedaquiline-based regimens, which showed 93.1% culture conversion at six months, is documented in a study [15]. Additionally, Borisov et al., in 2019, reported a 69.1% success rate in DR-TB patients undergoing adjunctive surgery, suggesting that surgery could improve treatment outcomes in select cases [16].

Treatment success also declined in Datia (from 75.65% in 2020 to partial recovery by 2022), with stable loss to follow-up rates (9%-10%). This mirrors findings of Jogdand et al. who linked treatment fluctuations to COVID-19 disruptions and healthcare access issues [12].

TB mortality increased in Datia (from 3.28% to 3.93%) and Tikamgarh (from 1.87% to 5.46%), consistent with findings of Singhal et al. (2024), who identified delayed diagnosis, treatment interruptions, and co-infections as key contributors to TB mortality [14]. HIV-TB co-infection rates increased in Datia (p = 0.07), highlighting the need for better integration of screening and treatment programs. A study conducted in South India found that 4.6% of TB patients were HIV-positive, with higher co-infection rates seen in pulmonary TB (7.2%) cases compared to EPTB (2.8%), indicating a significant burden in both new and previously treated TB cases [17]. Furthermore, another study revealed that HIV-TB co-infection weakens immune defenses due to high IL-10 secretion and PD1 expression, leading to poor antigen presentation, which may explain the higher TB mortality in co-infected individuals [18].

The limitations of this study include its retrospective design based on secondary data from the Nikshay portal, making the accuracy and completeness of the data subject to district-level reporting practices. The study did not account for socioeconomic determinants, comorbid conditions, or other variables that may influence TB incidence and outcomes. The data were not analyzed according to sex, occupation, and treatment adherence, limiting insights into specific high-risk groups. Variations in diagnostic and treatment practices across health facilities may have influenced the reported outcomes. The absence of qualitative data from healthcare providers and patients restricts a deeper understanding of the systemic challenges that affect TB control in these districts.

## Conclusions

Our study highlights significant trends in TB in the districts of Datia and Tikamgarh, which align with the national patterns in disease incidence, treatment outcomes, and associated risk factors. The observed reductions in pediatric TB and DR-TB indicate advancements in early diagnosis and treatment methods. However, the increasing burden of EPTB and the rise in notifications from the private sector point towards evolving epidemiological patterns and improvements in case detection. Despite these advancements, several challenges persist, including high mortality rates, declining treatment success, and stable loss-to-follow-up rates. These issues highlight the need for improved patient retention strategies and better access to healthcare services. The rise in HIV-TB co-infection emphasizes the necessity for integrated screening and treatment approaches. To strengthen TB control initiatives, it is essential to enhance engagement with the private sector, improve adherence programs, and expand diagnostic capabilities. The findings of this study may guide policy interventions aimed at achieving India’s TB elimination goals by addressing gaps in healthcare delivery and ensuring equitable access to high-quality treatment and care.

## References

[1] World Health Organization (2023). Global Tuberculosis Report 2023.

[2] Chandra K, Arora VK (2019). Tuberculosis and other chronic morbidity profile of sewage workers of Delhi. Indian J Tuberc.

[3] Kumar NP, Moideen K, Dhakshinraj SD (2015). Profiling leucocyte subsets in tuberculosis-diabetes co-morbidity. Immunology.

[4] Nagarajan K, Muniyandi M, Sellappan S (2023). A study on tuberculosis disease disclosure patterns and its associated factors: findings from a prospective observational study in Chennai. PLoS One.

[5] Nair D, Rajshekhar N, Klinton JS (2016). Household contact screening and yield of tuberculosis cases—a clinic based study in Chennai, South India. PLoS One.

[6] Htet KK, Liabsuetrakul T, Thein S, McNeil EB, Chongsuvivatwong V (2018). Improving detection of tuberculosis among household contacts of index tuberculosis patients by an integrated approach in Myanmar: a cross-sectional study. BMC Infect Dis.

[7] Saini V, Garg K (2020). Case finding strategies under National Tuberculosis Elimination Programme (NTEP). Indian J Tuberc.

[8] Sharma N, Bakshi R, Basu S, Zode M, Arora R, Khanna A (2023). Implementation of tuberculosis preventive therapy with INH-Rifapentine (3HP) for latent tuberculosis infection management in household tuberculosis contacts in India: a prospective study. Trop Med Int Health.

[9] Tripathy JP, Boelaert M, Van den Bergh R (2018). The impact of direct observation of treatment short-course (DOTS) on tuberculosis treatment outcomes: a systematic review. PLoS One.

[10] National TB Elimination Program. Overview of Sub-National Certification (SNC). https://ntep.in/node/4495/CP-overview-snc.

[11] Ni-kshay Reports. https://reports.nikshay.in/.

[12] Jogdand SJ, Bansode M, Gattani PL, Inamdar IF (2024). IJCM_256A: Trend in tuberculosis notification in Solapur district: analysis from 2015 to 2022. Indian J Community Med.

[13] Siddaiah A, Ahmed MN, Kumar AM, D'Souza G, Wilkinson E, Maung TM, Rodrigues R (2019). Tuberculosis notification in a private tertiary care teaching hospital in South India: a mixed-methods study. BMJ Open.

[14] Singhal L, Gupta P, Aysha K N, Gupta V (2024). Insights into changing patterns of extrapulmonary tuberculosis in North India. Indian J Med Microbiol.

[15] Mary Prince R, Khangarot S, Haque QF, Mittal A, Somani R, Grover M (2023). Outcomes of bedaquiline-containing regimen in the treatment of adults with drug-resistant tuberculosis in a tertiary care center in Rajasthan. Monaldi Arch Chest Dis.

[16] Borisov SE, D'Ambrosio L, Centis R (2019). Outcomes of patients with drug-resistant-tuberculosis treated with bedaquiline-containing regimens and undergoing adjunctive surgery. J Infect.

[17] Mohan A, Harikrishna J, Kumar DP, Kumar ND, Sharma PS, Kumar BS, Sarma KV (2017). Provider-initiated HIV testing & counselling in incident tuberculosis cases under National TB Programme conditions at a tertiary care teaching hospital in Tirupati, South India. Indian J Med Res.

[18] Ramaseri Sunder S, Suryadevara NC, Pydi SS, Neela VS, Valluri VL (2020). Defective antigen presentation leads to upregulation of PD1 and IL-10 in HIV-TB co-infection. J Interferon Cytokine Res.

